# A Novel Proposal for an Index for Regional Cerebral Perfusion Pressure – A Theoretical Approach Using Fluid Dynamics

**DOI:** 10.3389/fneur.2021.765463

**Published:** 2022-01-31

**Authors:** Masashi Kameyama, Toshimitsu Momose, Kenji Ishibashi, Kenji Ishii

**Affiliations:** ^1^Department of Diagnostic Radiology, Tokyo Metropolitan Geriatric Hospital and Institute of Gerontology, Tokyo, Japan; ^2^Department of Nuclear Medicine, Graduate School of Medicine, The International University of Health and Welfare, Narita, Japan; ^3^Research Team for Neuroimaging, Tokyo Metropolitan Geriatric Hospital and Institute of Gerontology, Tokyo, Japan

**Keywords:** positron emission tomography (PET), cerebral blood flow (CBF), cerebral blood volume (CBV), cerebral perfusion pressure (CPP), fluid mechanics

## Abstract

Cerebral blood flow (CBF) / cerebral blood volume (CBV) ratio derived by [^15^O] H_2_O/ CO_2_ and CO positron emission tomography (PET) examination has been used as an index for cerebral perfusion pressure (CPP). CBF/CBV was demonstrated to be related mean arterial pressure (MAP) in baboons. However, this formula has not been confirmed to be proportionate to CPP. We have developed a new index for CPP using the Poiseuille equation based on a simple model. Our model suggests that CBF/CBV^2^ is proportionate to CPP and that it is mathematically a more accurate index than CBF/CBV. This new index needs experimental validation in the future.

## Introduction

Cerebral perfusion pressure (CPP) is the driving force for cerebral blood flow (CBF) and, therefore, is an important factor for evaluation of a patient's cerebral hemodynamic state. However, a non-invasive method for measuring local CPP directly has yet to be developed.

As the CBF/cerebral blood volume (CBV) ratio derived by [^15^O] H_2_O/ CO_2_ and CO positron emission tomography (PET) examination reflected artery patency, CBF/CBV was proposed as an index for hemodynamic reserve (1). CBF/CBV was further found to be related to oxygen extraction fraction (2) and mean arterial pressure (MAP) in baboons (3). Based on these findings, CBF/CBV came to be used as an index for CPP (4, 5). When CPP decreases, CBV increases and CBF decreases, therefore, CBF/CBV certainly shows some relation to CPP. As CBF/CBV is the reciprocal number of mean transit time (6), it is proportionate to the mean velocity of blood. Certainly the fluid velocity falls according to the decrease in pressure (1–3).

Whilst CBF/CBV rises as CPP rises and vice versa, there is no evidence of ratio scale (i.e., there is no evidence that changes in CBF/CBV is proportional to changes in CPP). In this study, CPP was theoretically derived from CBF and CBV using fluid dynamics.

## Theory

**Figure 1:**
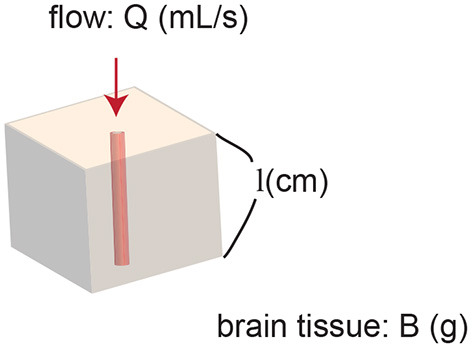
Model of a region of brain. The region contains one vessel. The vessel supplies blood flow to the entire region.

Assume there is one small cerebral region which contains one vessel. The vessel is the sole blood supply for the entire region (Figure 1).

The Poiseuille equation, which can be derived from the Navier-Stokes equations (7) describes incompressible fluid in lamina flow through a long pipe:


(1) $$\begin{array}{r} \Delta p=\frac{8\mu lQ}{\pi r^{4}} \end{array}$$

where Δ*p* denotes pressure difference between the two ends [i.e., local CPP (Pa)], μ is dynamic viscosity (Pa· min), *l* is length of the vessel (cm), *Q* is volumetric flow rate (mL/min), and *r* is radius of the vessel (cm).

CBF and CBV can be calculated as follows:


(2) $$\begin{array}{r} \text{CBF}=Q/B\;\left(mL/g/min\right) \end{array}$$

(3) $$\begin{array}{r} \text{CBV}=\pi r^{2}l/B\;\left(mL/g\right) \end{array}$$

where B denotes local brain tissue weight including the one vessel (g).

Therefore,


(4) $$\begin{array}{r} \text{CPP}=\frac{8\pi \mu l^{3}}{B}\frac{\text{CBF}}{\text{CB}{\text{V}}^{2}} \end{array}$$

As μ is constant and the volume of the brain region perfused by the single vessel is also approximately constant, CPP is proportional to CBF/CBV^2^.

Here, we would like to calculate cerebrovascular resistance (CVR).


(5) $$\begin{array}{r} \text{CBF}=\text{CPP}/\text{CVR} \end{array}$$

The Equation (1) can be arranged as follows using Equation (2):


(6) $$\begin{array}{r} \text{CPP}=\frac{8\mu lB}{\pi r^{4}}\frac{Q}{B}=\frac{8\mu lB}{\pi r^{4}}\text{CBF} \end{array}$$

Therefore,


(7) $$\begin{array}{r} \text{CVR}=\frac{\text{CPP}}{\text{CBF}}=\frac{8\mu lB}{\pi }\frac{1}{r^{4}} \end{array}$$

Hence, CVR is proportionate to 1/*r*^4^.

## Methods

### Simulation

A simulation was executed to demonstrate how CBF/CBV and CBF/CBV^2^ behave using a standard spreadsheet software, Excel (Microsoft Corporation, Redmond, WA, USA). It was run under the condition that the rate reduction of CBF per CPP after the auto-regulation limit [Powers' Stage II (8)] was twice the CBV elevation per CPP before the limit was reached (Stage I). The unit was a relative scale.

### Reanalysis of Mean Arterial Pressure and CBF/CBV^2^

CBF/CBV and CBF/CBV^2^ were calculated from CBF (mL/100mL/min) and CBV (mL/100mL) of the published study with baboons (3). The calculated values were plotted against MAP. Pearson's correlation coefficient and the linearity of the regression were assessed.

### Application to^15^O PET Study

One normal participant and one patient with Moyamoya disease were analyzed. The procedure of^15^O PET study was documented in Hara et al. (9). Both participants were scanned with Discovery 710 PET/CT system (GE Healthcare, Milwaukee, WI, USA).

## Results

### Simulation

**Figure 2:**
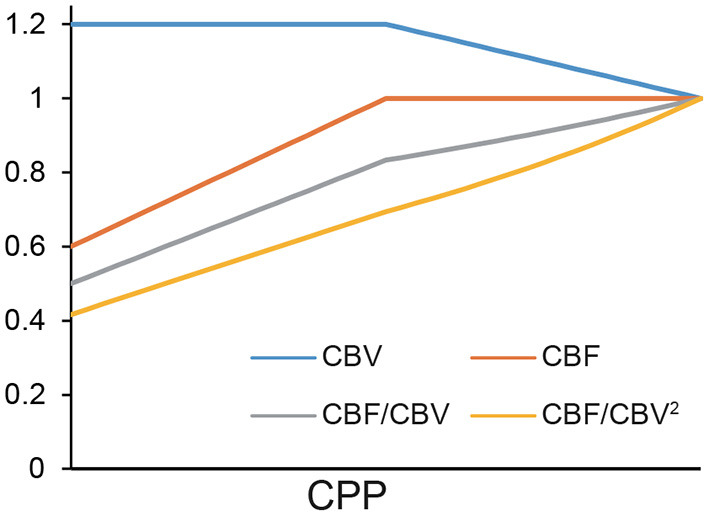
A simulation of CBF/CBV and CBF/CBV^2^.

CBF/CBV^2^ showed better linearity than CBF/CBV (Figure 2).

### Reanalysis of MAP and CBF/CBV^2^

**Figure 3:**
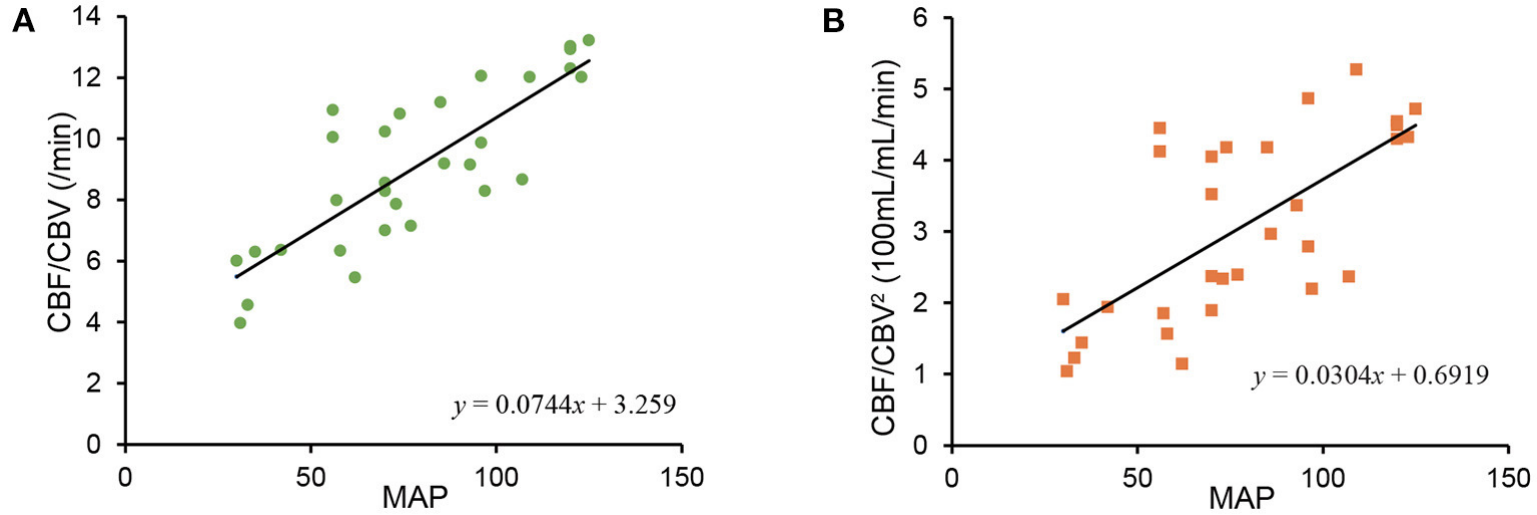
Relationship between mean arterial blood pressure (MAP) and CBF/CBV **(A)**, between MAP and CBF/CBV^2^ **(B)**.

CBF/CBV and CBF/CBV^2^ were plotted against MAP (Figure 3). Both CBF/CBV and CBF/CBV^2^ showed significant correlation with MAP (CBF/CBV *r* = 0.8229, *p* = 2.398 × 10^−8^; CBF/CBV^2^ *r* = 0.6833, *p* = 3.157 × 10^−5^). Regression lines were CBF/CBV = 0.0744 MAP + 3.259 and CBF/CBV^2^ = 0.0304 MAP + 0.6919. CBF/CBV showed better correlation coefficient, however, it showed larger intercept.

### Application to^15^O PET Study

**Figure 4:**
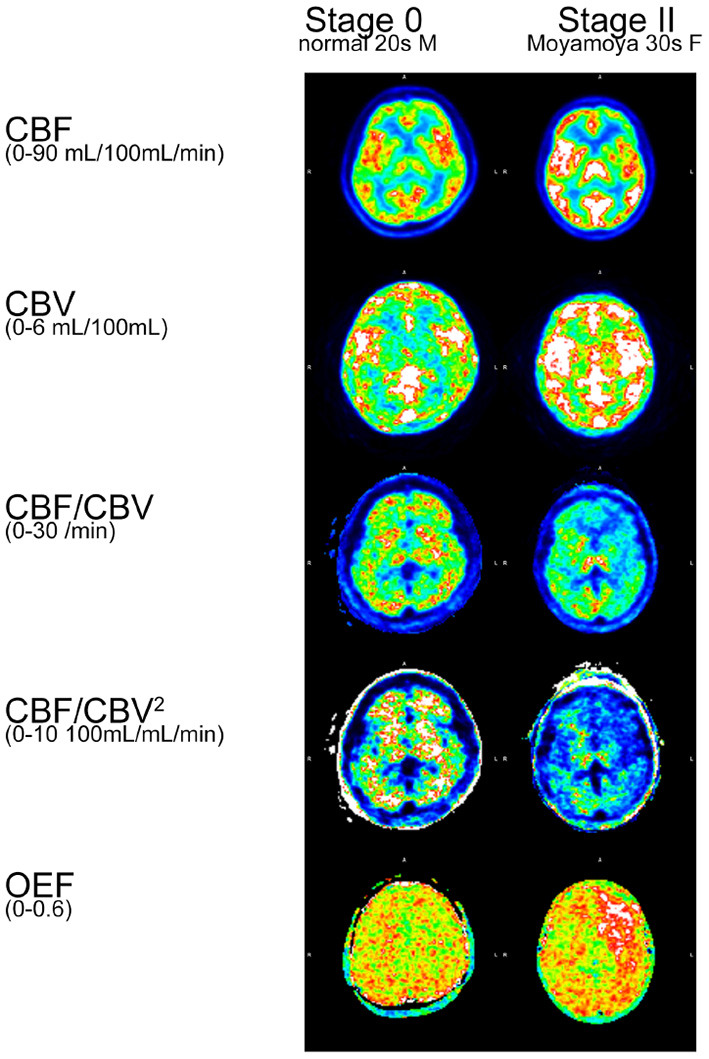
Example of^15^O PET study.

CBF, CBV, CBF/CBV, CBF/CBV^2^, OEF were calculated for a normal participant and a patient with Moyamoya disease (Figure 4). CBF/CBV^2^ showed pronounced decrease in entire brain of patient with Moyamoya disease.

## Discussion

We have demonstrated theoretically that CBF/CBV^2^ is an appropriate indicator for CPP. CBF/CBV is the reciprocal number of mean transit time (6). Therefore, it is proportionate to the mean velocity of blood, and certainly relates to CPP. However, our theoretical approach implies that CBF/CBV^2^ would be a better approximation for CPP than CBF/CBV.

The linearity shown in Figure 2 was determined in part by our assumption of CBF reduction being twice that of CBV elevation. However, this is a reasonable assumption considering the fluid dynamics equations above. Within auto-regulation limit (Stage I), Equations (1, 3) tells that CBV is proportionate to CPP^−0.5^, therefore, ΔCBV $≃k\left(-\frac{1}{2}\right)\Delta$CPP (*k*: constant). In Stage II, Equations (1, 2) tells that CBF is proportionate to CPP.

Figure 2 showed small difference between CBF/CBV and CBF/CBV^2^. This simulation confirmed CBF/CBV as an index for CPP although it is not a ratio scale.

Reanalysis of MAP and CBF/CBV, CBF/CBV^2^ (Figure 3) showed less stability of CBF/CBV^2^. The instability may be attributable to spill over from [^15^O] CO in venous and physiologically small proportion of CBV (about 4% of brain tissue). As CBV being small is difficult to measure with accuracy, CBF/CBV^2^ may not be reliable comparing to CBF/CBV in a physical world. However, regression line of CBF/CBV^2^ passed near origin, which would demonstrate potential superiority.

Calculated CBF/CBV^2^ images (Figure 4) showed pronounced decrease in entire brain of patient with Moyamoya disease, comparing with CBF/CBV images. Considering serious prognosis of Moyamoya disease, CBF/CBV^2^ images would reflect the status of the disease. Furthermore, the figure showed that clearly elevated OEF in the area of low CBF/CBV^2^, which is consistent with the well established notion that high OEF is a very sensitive index of lost autoregulation (10).

The Poiseuille equation is applicable under the conditions of laminar flow in a long tube. Thus, our conclusions may not be applicable in situations of turbulent flow. However, the effect of turbulent flow are likely limited as Reynolds number $\left(\frac{2r\rho v}{\mu }\right)$ (*v*: velocity of fluid, ρ: the density of the fluid) of small vessel is small (capillary: 0.0007–0.003, arteriole: 210–570, Reynolds number smaller than 2300 indicates laminar flow).

This new index needs experimental validation in the future.

## Data Availability Statement

The original contributions presented in the study are included in the article/supplementary material, further inquiries can be directed to the corresponding author/s.

## Author Contributions

MK contributed to the conceptualization, creation of theory, and initial draft manuscript preparation. TM advised the project. KIshib and KIshii contributed the calculation of^15^O PET studies. All the authors discussed the project and have read and approved the final manuscript.

## Conflict of Interest

The authors declare that the research was conducted in the absence of any commercial or financial relationships that could be construed as a potential conflict of interest.

## Publisher's Note

All claims expressed in this article are solely those of the authors and do not necessarily represent those of their affiliated organizations, or those of the publisher, the editors and the reviewers. Any product that may be evaluated in this article, or claim that may be made by its manufacturer, is not guaranteed or endorsed by the publisher.
